# RAVAN: CubeSat Demonstration for Multi-Point Earth Radiation Budget Measurements

**DOI:** 10.3390/rs11070796

**Published:** 2019-04-03

**Authors:** William H. Swartz, Steven R. Lorentz, Stergios J. Papadakis, Philip M. Huang, Allan W. Smith, David M. Deglau, Yinan Yu, Sonia M. Reilly, Nolan M. Reilly, Donald E. Anderson

**Affiliations:** 1 Johns Hopkins University Applied Physics Laboratory, Laurel, MD 20723, USA; 2 L-1 Standards and Technology, Manassas, VA 20109, USA

**Keywords:** Earth radiation budget, outgoing longwave radiation, reflected solar radiation, energy imbalance, carbon nanotubes, gallium black body, CubeSat

## Abstract

The Radiometer Assessment using Vertically Aligned Nanotubes (RAVAN) 3U CubeSat mission is a pathfinder to demonstrate technologies for the measurement of Earth’s radiation budget, the quantification of which is critical for predicting the future course of climate change. A specific motivation is the need for lower-cost technology alternatives that could be used for multi-point constellation measurements of Earth outgoing radiation. RAVAN launched 11 November 2016, into a nearly 600-km, Sun-synchronous orbit, and collected data for over 20 months. RAVAN successfully demonstrates two key technologies. The first is the use of vertically aligned carbon nanotubes (VACNTs) as absorbers in broadband radiometers for measuring Earth’s outgoing radiation and the total solar irradiance. VACNT forests are arguably the blackest material known and have an extremely flat spectral response over a wide wavelength range, from the ultraviolet to the far infrared. As radiometer absorbers, they have greater sensitivity for a given time constant and are more compact than traditional cavity absorbers. The second technology demonstrated is a pair of gallium phase-change black body cells that are used as a stable reference to monitor the degradation of RAVAN’s radiometer sensors on orbit. Four radiometers (two VACNT, two cavity), the pair of gallium black bodies, and associated electronics are accommodated in the payload of an agile 3U CubeSat bus that allows for routine solar and deep-space attitude maneuvers, which are essential for calibrating the Earth irradiance measurements. The radiometers show excellent long-term stability over the course of the mission and a high correlation between the VACNT and cavity radiometer technologies. Short-term variability—at greater than the tenths-of-a-Watt/m^2^ needed for climate accuracy—is a challenge that remains, consistent with insufficient thermal knowledge and control on a 3U CubeSat. There are also VACNT–cavity biases of 3% and 6% in the Total and SW channels, respectively, which would have to be overcome in a future mission. Although one of the black bodies failed after four months, the other provided a repeatable standard for the duration of the project. We present representative measurements from the mission and demonstrate how the radiometer time series can be used to reconstruct outgoing radiation spatial information. Improvements to the technology and approach that would lead to better performance and greater accuracy in future missions are discussed.

## 1. Introduction

Climate change is driven by a small yet crucial net imbalance in the Earth’s global energy budget [1–7]. This Earth energy imbalance (EEI) is manifested at the top of the atmosphere (TOA) as the difference between the incoming solar energy reaching the Earth (total solar irradiance (TSI)) and the outgoing solar-reflected and thermally emitted energy (Earth outgoing radiation (EOR)). Current research suggests that the imbalance is less than +1 out of 340 W/m^2^ on an annual global mean basis (meaning that the outgoing energy is less than the incoming solar energy by about 0.3%) (e.g., [8]), due to the net radiative forcing of anthropogenic emissions of greenhouse gases and aerosols [9].

The TSI has been measured continuously from space since the 1970s [7]. The absolute TSI value from the Total Irradiance Monitor (TIM) on the SOlar Radiation & Climate Experiment (SORCE) is 1360.8 ± 0.5 W/m^2^ at solar minimum [10]. By combining the most reliable, independent TSI-measuring instruments, Dewitte and Clerbaux derived a value of 1362.0 ± 0.9 W/m^2^ [7]. Achieving the current low level of absolute uncertainty has required a significant long-term effort but is benefitted by the fact that the Sun is a reasonably stable light source, occupying a small, 0.5° angular extent at the distance of Earth’s orbit. Measuring the outgoing radiation has additional challenges in that it spans not only solar-reflected wavelengths but also the Earth’s radiative emission, which extends far into the thermal infrared. The outgoing radiation arrives at a spacecraft in low Earth orbit from a larger angular extent close to 120°, and there is tremendous spatial and temporal variability that cannot be sampled simultaneously by a single or small number of sensors, with profound implications for sampling bias and instrument inter-calibration.

Space-based measurements of EOR have been made nearly continuously since the 1970s [7,11], similar to TSI. These comprise instruments with scanning, narrow field-of-view (NFOV) and non-scanning, wide field-of-view (WFOV) configurations, the latter viewing the entire Earth disk at once. In either case, radiometers with and without filters blocking thermal wavelengths are used to distinguish the shortwave (SW) solar-reflected and longwave (LW) terrestrial emission components of the upwelling flux. The WFOV nonscanners are essentially single-pixel sensors and thus are unable to resolve radiance spatial variation within an individual FOV natively, although spatial information may be reconstructed from time series measurements along the orbit track [12–14]. In contrast, the NFOV instruments scan the visible Earth disk with FOVs of about 10–100 km, with varying viewing geometries. An important distinction between the WFOV and NFOV approaches is that the former measures the flux at satellite altitude, whereas the latter measures radiance from within each NFOV. In the latter case, the flux is estimated by integrating the NFOVs assuming an angular dependency of the radiation field based on the scene type within the NFOV [15]. The Earth Radiation Budget Experiment (ERBE) [16] and Clouds and the Earth’s Radiant Energy System (CERES) [17] series of instruments in low Earth orbit represent a long EOR data record back to the 1980s, although not without interruption. These instruments, in Sun-synchronous polar orbits, achieve global coverage but lack independent temporal sampling. The Geostationary Earth Radiation Budget (GERB) instruments [18] have diurnal sampling but individually view only about one third of Earth longitudes and with only oblique sampling at high latitudes. A combination of ERB scanning instruments leads to an EOR value of 336.1 ± 4.7 W/m^2^ [7], with an absolute uncertainty limited by calibration, inter-calibration, and sampling.

The Earth energy imbalance mentioned above may be calculated from the extant space-based TSI and EOR measurements listed above, EEI = TSI/4 EOR, leading to an EEI of 4.3 ± 4.9 W/m^2^ (note that TSI must be divided by 4 to compute the incoming solar flux arriving at the global TOA). This is quite different from the value derived independently from ocean heat content measurements: 0.9 ± 0.3 W/m^2^ [19]. Current space-based measurements of EOR have good long-term stability and thus are critical for tracking changes in EEI [20], but the level of uncertainty is too large to adequately quantify the absolute value of EEI with the needed “climate accuracy” of tenths of a W/m^2^ (or about 0.1% of the roughly 340 W/m^2^ EOR signal) (e.g., [2,21]).

As a next-generation ERB concept, Wiscombe and Chiu proposed a space-based constellation of sensors that would be able to overcome the sampling limitations and provide global, diurnal measurements of EOR [22]. With advances in radiometer and small satellite technologies [23], it is possible to envision a network of small space-based sensors that could measure the Earth’s highly variable outgoing radiation field. Gristey et al. showed how a notional EOR constellation of WFOV broadband radiometers can recover both spatial and temporal information [14]. Improved sampling notwithstanding, Wong et al. have recently argued that the calibration and inter-calibration of such a constellation is currently an intractable challenge, based on experience with the ERBE WFOV instruments [24].

The RAVAN CubeSat project is a technology demonstration to prove two technologies that could enable an ERB constellation and/or play a role in the next generation of ERB measurement technologies: radiometers with vertically aligned carbon nanotube (VACNT) absorbers and gallium phase-change black body calibration sources [25–28]. Vertically aligned carbon nanotube “forests” are some of the lowest-reflectivity (blackest) materials known and have an extremely flat spectral response over a wide wavelength range. The second key technology is the gallium calibration source. Two gallium fixed-point black bodies serve as on-orbit infrared sources that, when coupled with deep-space looks, provide an additional means to determine the radiometer gains. We use the gallium solid–liquid phase transition (29.76°C) as a stable, repeatable reference for the black body emission to track the long-term degradation of the radiometers.

The RAVAN payload is flown on a 3U CubeSat. The CubeSat provides a convenient, relatively inexpensive platform to raise the technology readiness of the new RAVAN technologies. As a future ERB constellation would necessarily be driven to use smaller spacecraft to minimize costs, the CubeSat demonstration allows us the opportunity to explore the strengths and weaknesses of such a small satellite approach. RAVAN is a technology demonstration, and it does not solve the outstanding calibration challenges raised by Wong et al. [24]. It also is not intended to provide continuity of the climate data records established by NFOV instruments such as CERES. Rather, it serves as a benchmark for future ERB science missions that may use RAVAN technologies and/or smaller spacecraft.

We describe the design and implementation of VACNT-based broadband radiometers and accompanying gallium black bodies in Section 2. In Section 3, we assess the performance of the technologies during a 20-month experiment in space, including calibration, measured EOR at spacecraft altitude, and the identification of observed geophysical features, as well as a sample reconstruction of LW flux spatial information using spherical harmonic fitting of the RAVAN WFOV time series. The discussion in Section 4 puts the strengths and weaknesses of the RAVAN mission in the context of the measurement we are attempting and describes ways in which the approach could be improved in future missions.

## 2. Materials and Methods

Broadband radiometers are typically used to measure the total energy input to or output from the Earth [16,17,29]. The ideal radiometer receiver is a perfect absorber across the spectrum and has very little thermal mass. The latter characteristic allows for faster light detection or conversely more sensitive detection for a given time constant. Traditionally this has meant a conical cavity with a black-painted interior where photons are absorbed within several bounces and before escaping the cavity. Carbon nanotubes (CNTs) are arguably the blackest material known and are used to make incredibly black coatings [30]. VACNT forests are the ideal absorbing material for a radiometer receiver: they enable construction of a spectrally black receiver with a single absorbing surface that results in a lower thermal mass than a receiver of a cavity design.

The goal for RAVAN is to demonstrate the use of VACNTs as broadband radiometer absorbers in space. One question is whether the CNTs degrade in a space environment due to a combination of spacecraft outgassing and ultraviolet (UV) exposure. To answer that question, cavity radiometers, which are inherently insensitive to such degradation, are included on the RAVAN payload for comparison. Changes in the ratio of the cavity and VACNT detectors when both look at the Sun and separately at the Earth can provide insight into the spectral nature of any VACNT degradation.

In addition to demonstrating RAVAN’s essential technologies, we endeavored to make the most stable and accurate measurements possible in a 3U CubeSat form factor (10 × 10 × 34 cm^3^), with an accuracy goal of 0.3 W/m^2^. The basic approach is to treat EOR as a simple irradiance measurement, employing thermal detectors with a black and spectrally flat absorber and precision aperture. For RAVAN this resulted in a set of four broadband radiometers that measure the temperature changes of an absorber exposed to radiant flux from a FOV defined by an aperture and baffle. The radiometer absorbers comprise either a 2-dimensional VACNT forest or traditional black-painted cavity.

The payload requirements for RAVAN are summarized in Table 1. First, a stable black body emitter is used to monitor degradation and provide for calibration transfer to pre-launch calibration. Gallium, which melts at 29.76 °C, is well suited for this purpose. A gallium black body, coupled with solar and space looks, gives offset and degradation monitoring. The payload design also requires adequate resolution and absolute scale to determine EOR.

We originally planned an extensive pre-launch ground calibration, including piece-part characterization of the aperture area and land scatter, spectral reflectance of the cavities, spectral transmission of the sapphire domes; subassembly characterization of the aperture land scatter, scattered light inside the domes, scattered light from the FOV baffle, angular response through the sapphire domes; and completed radiometer laser power mode responsivity, angular response function, and irradiance response as a function of FOV. Due to the vagaries of getting a CubeSat to space as a secondary payload, not least because of our requirement to be above 550 km altitude, we elected to take an unanticipated launch opportunity with a compressed launch schedule. This meant that much of our ground calibration would have to be eliminated, and instead we would have to rely on on-orbit calibration. This was advantageous for a timely technology demonstration but made absolute calibration much more difficult. The Sun would therefore be our source for absolute scale.

On-orbit calibration is described in more detail in Section 2.3, but briefly, the following modes are needed. Solar calibration provides the absolute radiometric scale, with cold deep-space calibration providing the offset. Gallium black body calibrations provide a stable reference on orbit to monitor changes in the radiometers. Inter-calibration of the primary and secondary radiometers (VACNT vs. cavity) provides another important basis for comparison and degradation monitoring. Finally, internal calibration, where a constant amount of energy is input directly into the radiometer absorbers, removing the thermal link, thermistors, and bridge circuit from the calibration.

### 2.1. Broadband Radiometers

**Figure 1:**
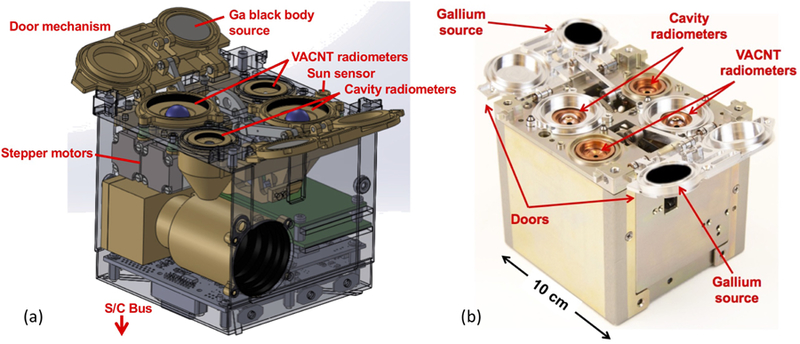
RAVAN payload. (**a**) Mechanical prototype. (**b**) Photograph of the flight payload (note: photograph turned 180 compared to the model). The payload is contained within a 1U volume (10 × 10 × 10 × cm^3^) and sits atop the spacecraft (s/c) bus (not shown).

The RAVAN payload houses primary and redundant pairs of radiometers, as well as a pair of gallium reference sources integrated into two motor-driven doors, as illustrated in Figure 1. The primary radiometers use VACNT absorbers, while the redundant pair (the secondary radiometers) use black-painted cavity absorbers. Each radiometer pair includes a total (Total) channel whose spectral band is set by the absorber and sensitive to nearly all EOR, from the UV to the far infrared (IR), and a SW channel whose band is limited by a sapphire dome, nominally sensing reflected solar radiation. The radiometers, listed in Table 2, are actively temperature controlled and thermally isolated from the spacecraft. The reusable doors open wide enough to clear the 135° FOVs of the radiometers and close to protect the radiometers when they are not being used. Custom radiation-tolerant electronics control the payload, collect data, and communicate with the spacecraft.

#### 2.1.1. Vertically Aligned Carbon Nanotube (VACNT) Absorbers

**Figure 2:**
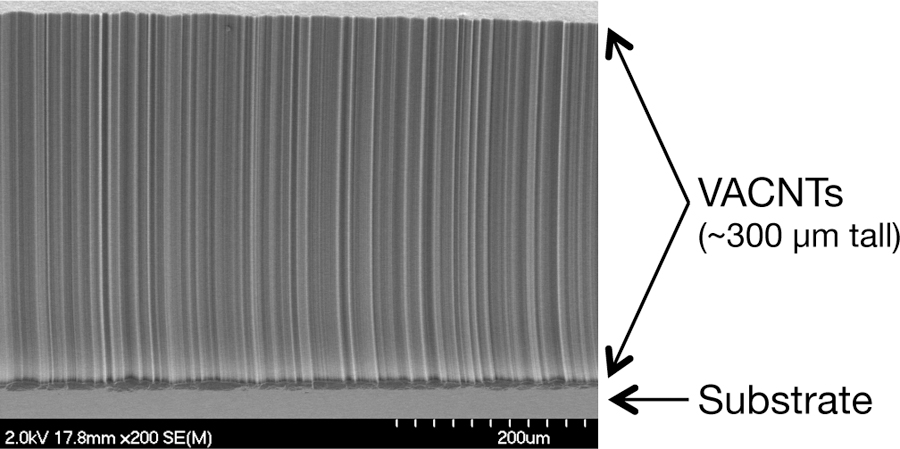
Scanning electron micrograph of a Vertically Aligned Carbon Nanotube (VACNT) forest, similar to those flown on RAVAN. The image is an edge-on view or cross section of the forest.

Carbon nanotubes are an allotrope of carbon that, at a microscopic level, are essentially long, hollow graphene cylinders [31]. These nanostructures have several unusual properties that make them ideal for certain applications, including stray light absorption on baffles and blankets, pyroelectric sensors, heaters, and, particularly, bolometer and radiometer sensor heads. VACNT forests grow spontaneously under the appropriate laboratory conditions and are mostly (99.9%) empty space (see Figure 2), which contributes to their facility in capturing photons. VACNT forests have enough carbon nanotube interconnections that they are mechanically robust and do not cause particulate contamination. They are promising as radiometer absorbers because they are compact, have a very large thermal conductivity, and have greater sensitivity for a given time constant than comparable cavity radiometers. Also, because VACNTs are pristine ordered carbon surfaces, they do not outgas, making them ideal for space-based applications.

**Figure 3:**
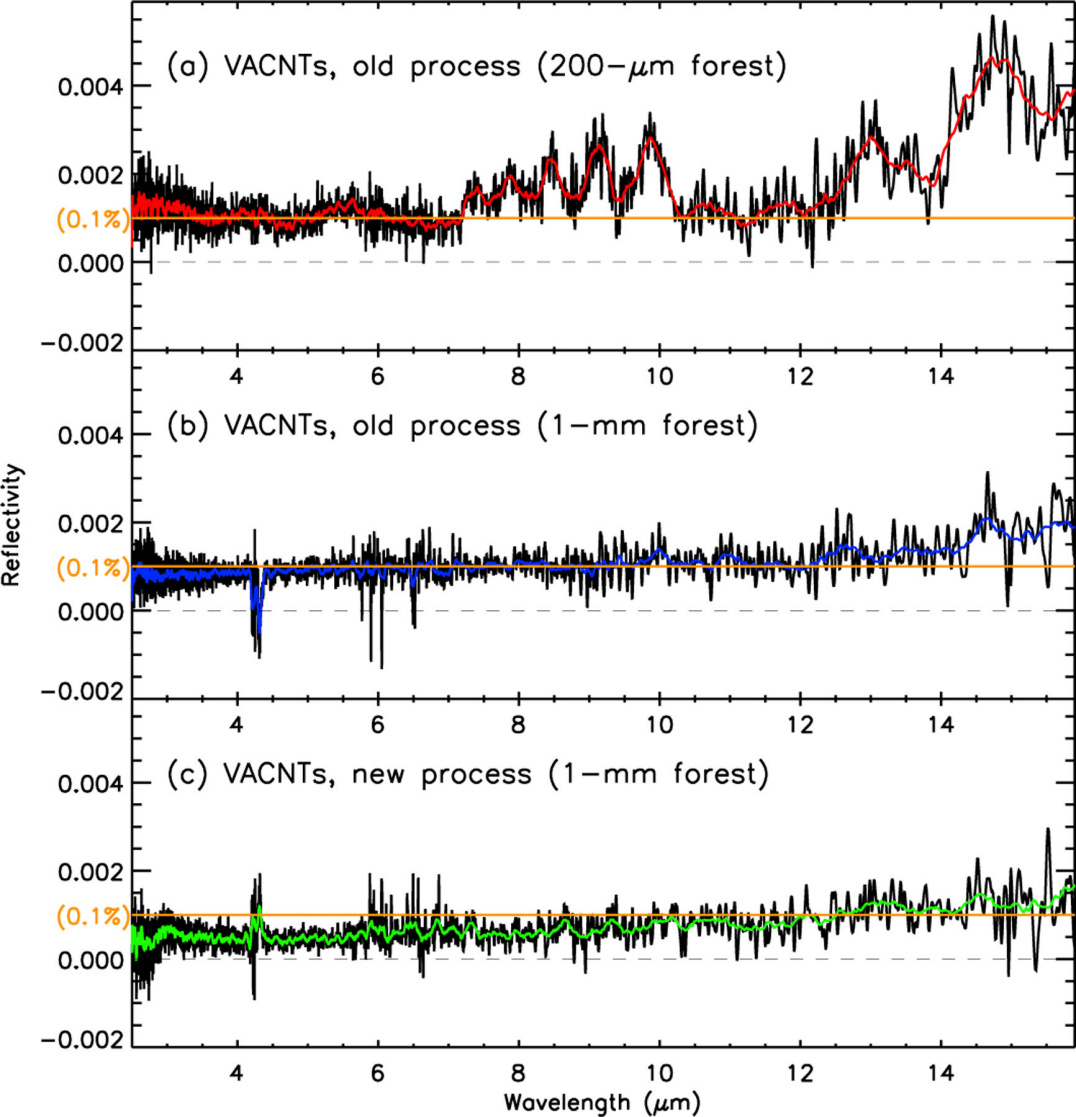
VACNT reflectivity as measured at APL for several growth processes and post-growth modifications. The 0.1% reflectivity target is indicated with horizonal orange lines in each panel. The colored (red, blue, green) lines are the raw data (black) smoothed over wavelength. (**a**) Initial process: 200-µm-tall VACNT forest. (**b**) 1000-µm (1-mm)-tall forest. (**c**) 1000-µm-tall forest growth, with the addition of aggressive post-growth oxygen plasma etching.

The VACNTs used in RAVAN’s radiometers were grown at the Johns Hopkins University Applied Physics Laboratory by a process described in Appendix A. The growth and post-growth treatment procedure was developed over time and refined for RAVAN. Our reflectivity target is 10^−3^. For RAVAN we generated a library of VACNT growths (and post-growth treatments) and then selected the best performing (blackest out to 16 µm). Figure 3 shows the progress made toward our reflectivity goal. Our initial attempts, with a 200-µm tall forest, were not satisfactory. We experimented with forests of different heights and with multiple stages of growth (where an internal discontinuity along the CNTs is introduced by stopping and restarting the carbon gas flow mid-growth), and we found that the best results were obtained with a 1000-µm (1-mm) growth. Taller growths were not practical given the internal geometry of the radiometer heads. The 1-mm VACNTs barely met our reflectivity target, so we turned to the post-growth treatment. We employed an aggressive oxygen plasma etch [32]. This resulted in reflectivities below 10^−3^ at wavelengths out to 13 µm. Incidentally, we found that the aggressive oxygen etch was less effective for “blackening” the initial, shorter VACNT forests.

### 2.1.2. Radiometer Design

The broadband radiometers employed in RAVAN are effectively linear-response thermal detectors that sense the heating of an optical absorber, the receiver, by incident radiation. The resulting change in the receiver temperature is measured electronically with a thermistor. Aside from degradation of the absorber, changes in the thermistor, its readout electronics, or the thermal impedance between the receiver and its heatsink will alter the detector response. To remove these effects a calibration heater is placed on each receiver to monitor and correct for such changes during the mission (this also linearizes the response over significant changes in operating temperature). The stability of individual detectors is established using both the heaters and by viewing sources—either the Sun, which is itself essentially stable, or the Earth simultaneously with a cavity radiometer that acts as a reference.

Each radiometer receiver is mounted to its own temperature-controlled heatsink, which provides a stable thermal environment as the spacecraft temperature cycles by approximately 20 °C within the payload during an orbit. In addition to reducing the resulting changes in the background, temperature control of the heatsink simplifies calibration of the radiometers, whose sensitivity depends on temperature due primarily to the thermistor.

The VACNT and cavity radiometers have many common design elements including the sapphire domes of the SW channels as well as a defining detector aperture and a baffle that limit the FOV. In addition, both radiometers employ a printed circuit board (PCB) that supports the receivers, provides a controlled thermal link to the heatsink, and holds the heaters and thermistors. The PCB attaches to the heatsink and receivers using solder. The heatsink heaters and thermistors reside on the PCB and protrude into pockets milled into the heatsink. The pockets are filled with epoxy to maximize thermal contact to the heatsinks. There are four thermistors wired together that collectively sense the heatsink temperature while minimizing the effects of temperature gradients. To facilitate soldering to the PCBs, the silicon chips holding the VACNTs are metalized (with gold—see Appendix A) on one side. The receiver cavities, which are made of gold-plated electroformed copper, are soldered into the PCB before being painted with Aeroglaze Z302 paint. Receiver thermistors and heaters of the VACNT radiometers are soldered directly to the PCB on the opposite side of the silicon chip. For the cavity radiometers they are glued to the cavity and wired to pads located on the PCB.

**Figure 4:**
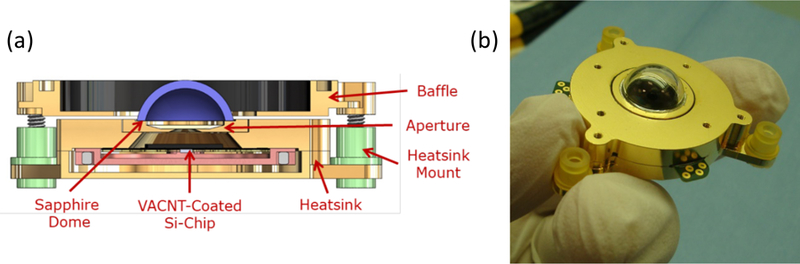
VACNT radiometer. (**a**) VACNT shortwave radiometer solid model cross section. (**b**) Photograph of assembly without the baffle, prior to integration.

**Figure 5:**
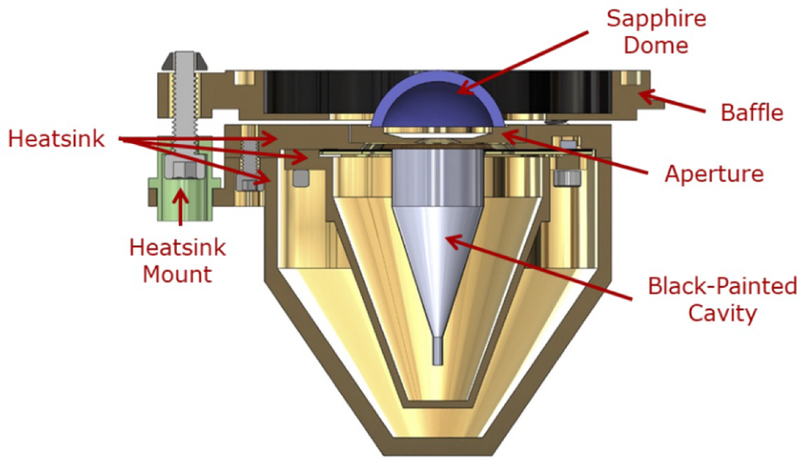
Cavity shortwave radiometer solid model cross section.

Figure 4 shows a cross section of the VACNT shortwave radiometer along with a photograph of the detector prior to integration. The heatsink mounts provide thermal isolation from the spacecraft; they are made of Ultem®—a very strong plastic with low thermal conductivity. The sapphire dome is soldered to the heatsink and maintains a stable longwave infrared background. Sapphire was chosen over quartz for its high thermal conductivity, which minimizes temperature gradients. The analogous cavity SW radiometer is shown in Figure 5.

Details of the temperature sensing and electronics used in RAVAN’s radiometers are found in Appendix B.

### 2.2. Gallium Black Bodies

In addition to celestial sources, RAVAN employs a gallium phase-change black body to monitor detector stability at long wavelengths. The design employed on RAVAN is intended as a stability monitor rather than as an absolute source due to limited space and power. A larger cavity design would provide a very high-emissivity radiator whose temperature is very near the gallium melting point of 29.76 °C. Instead, a much more compact design using a VACNT-on-Si emitting surface that fits into the radiometer doors is used. A goal of the mission is to establish that the gallium black bodies provide a stable on-orbit radiometric reference as the gallium undergoes melt/freeze transitions.

**Figure 6:**
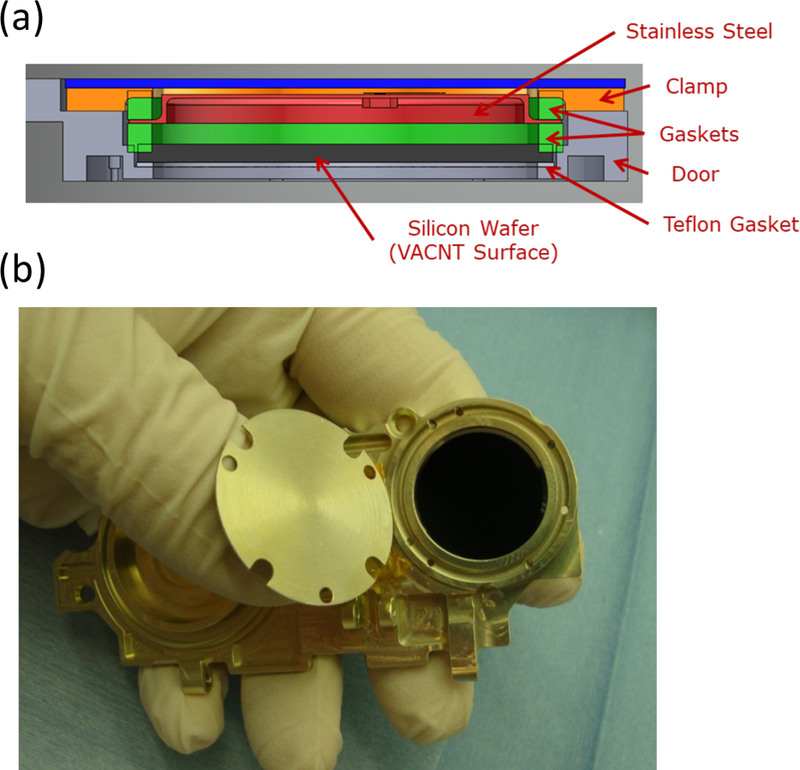
Gallium black body. (**a**) Solid model cross section of one of the two gallium black body reference sources that are integrated into each door (light gray). The gallium is contained by a 1-mm thick silicon wafer (dark gray), two silicone rubber gaskets (green), and a stainless steel cover (red). An aluminum clamp (orange) compresses the two gaskets; the lower forms a seal between the cover and silicon wafer, while the upper allows for vertical expansion of the contained volume during a freezing transition. A Teflon gasket prevents fracture of the brittle silicon wafer due to irregularities in the mating surface and provides some thermal isolation from the doors. (**b**) Photograph of a detached door showing the emitting side of a gallium black body reference source. Please note that the photograph contains a circular temporary cover used to protect the VACNT surface prior to integration. The VACNT emitter (black disk) that is about 1 inch in diameter.

Each door contains an integral gallium black body that acts as a long wavelength radiometric source and is positioned over the Total channel radiometer. Pure gallium (4.25 g) is enclosed in each cell, shown in Figure 6, which consists of a silicon wafer, the VACNT-coated emitter, a stainless steel top, and two silicone rubber gaskets that create seals against the silicon and stainless surfaces. An aluminum flange compresses the gaskets to contain the gallium. Stress analysis was performed to verify that the silicon wafer can withstand the internal pressure increase due to the expansion of the gallium as it freezes. The cells’ highly compressible silicone rubber gaskets easily accommodate the 3% expansion of gallium upon freezing.

The emitted spectrum is expected to approximate an ideal black body, as the VACNTs grown on the silicon surface facing the Total channel radiometer are good absorbers (see Section 2.1.1) and therefore good emitters. However, temperature gradients within the cell and less-than-unity emissivity preclude its use as a low-uncertainty absolute source. A thermistor and heater integrated into the stainless steel cover provide a monitor of the phase transition and a means to actively melt the gallium. The materials that are in contact with the gallium, i.e., silicone rubber, silicon, and stainless steel, all resist corrosion at the relatively low temperatures encountered during the mission. By contrast, aluminum reacts strongly when in contact with liquid gallium at room temperature and becomes embrittled.

**Figure 7:**
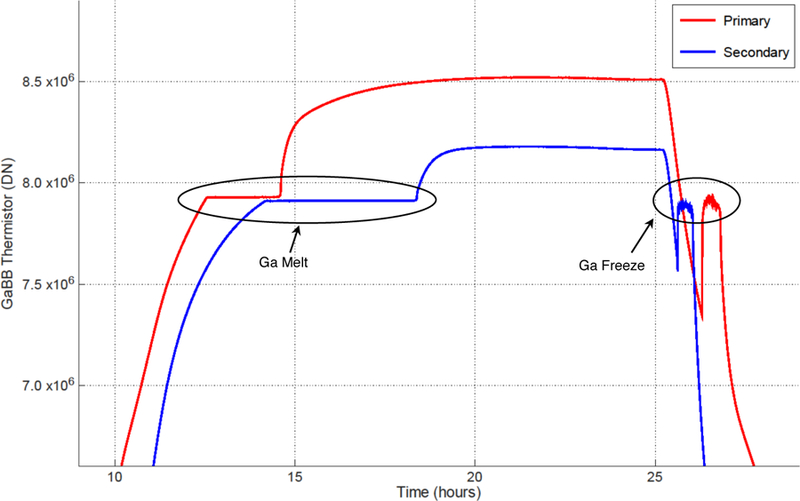
Gallium cell transitions during thermal vacuum testing. The gallium cell thermistor readings are shown as a function of time for each cell. Melting and freezing transitions (see labels) correspond to time periods where temperatures are relatively constant with time.

Gallium black body transitions during thermal vacuum testing are shown in Figure 7. The melt is well behaved, the freeze less so although not unexpectedly. Similar behavior is seen on orbit (see Section 3.1). There are at least two explanations for the complex freezing transition. The first is that the gallium, gallium cell housing, and thermistor are not in perfect thermal contact, and it is possible that the beginning of the freeze is not communicated well to the thermistor, allowing the thermistor to cool below the freezing gallium. Another possibility is that the gallium supercools prior to freezing. This behavior has been noted previously. Topham et al. experienced gallium supercooling in their on-orbit International Space Station experiment evaluating the effect (if any) of microgravity on gallium phase changes [33]. In any case, gallium melts were used exclusively for RAVAN radiometer stability measurements; the freezing behavior had no effect.

### 2.3. On-Orbit Operations

**Figure 8:**
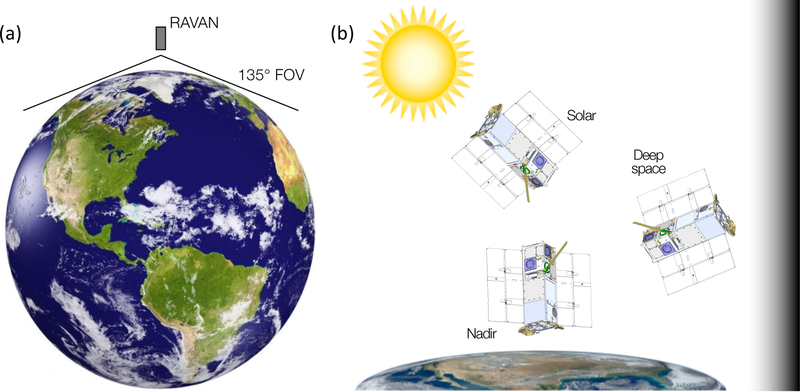
RAVAN concept of operations. (**a**) Nadir view, at an altitude high enough (>550 km) for the Earth disk to subtend the wide FOV. (**b**) Attitudes supporting normal (nadir) operations and various calibration maneuvers (see also Table 3).

The RAVAN payload was integrated with a 3U CubeSat bus built by Blue Canyon Technologies, their model XB1. The RAVAN XB1 has 3-axis attitude determination and control (at the level of 10 arc-seconds over the course of several minutes during stare maneuvers) and UHF communications. RAVAN’s basic concept of operations is shown in Figure 8. As designed, RAVAN could operate continuously day and night and downlink all telemetry. The typical configuration is nadir Earth viewing, with the Earth disk subtending the wide FOV with an approximately 1° margin. Data analysis would have been significantly less certain if we had had to model any part of the Earth disk falling outside the FOV. In the normal nadir configuration, only the VACNT radiometer door is open; the cavity door is typically closed except during inter-calibration. The rationale for this was to reduce the exposure of the cavity radiometers and to monitor degradation.

Normal nadir operations are interrupted by calibration maneuvers, as summarized in Table 3 (and Figure 8b). Direct solar views provide the absolute radiometric scale, with cold deep-space views providing the offset. The solar calibrations comprise both a period of staring at the Sun, with the Sun in the center of the FOV, and another period of dwells at a series of solar angles ranging from −45° to +45°. This was to measure the angular response functions of the detectors on orbit (see Appendix C). Gallium black body calibrations provide a stable reference on orbit to monitor changes in the radiometers. Inter-calibrations of both the primary and secondary radiometers provide another important basis for comparison and degradation monitoring. The CubeSat bus provides all necessary attitude control for the calibration maneuvers. In practice, all the calibration operations are contained in a single day. Our pre-launch plan was to execute calibration sequences weekly to monthly, depending on the rate of changes on orbit. Due to communications challenges we experienced during the mission, however, we typically alternated between nadir-viewing and calibration days, to ensure sufficient opportunities for both.

## 3. Results

RAVAN was launched 11 November 2016, as a secondary payload on an Atlas V rocket from Vandenberg Air Force Base in the state of California, USA, into a nearly 600-km, nearly circular Sun-synchronous orbit with an Equator crossing time of about 10:30 AM for the descending node. From a technology demonstration standpoint, the RAVAN mission ended 1 August 2018 (RAVAN day 628), but the spacecraft continues to operate as of this writing. In this section, we describe data collected during the mission and their interpretation.

### 3.1. Gallium Black Bodies

**Figure 9:**
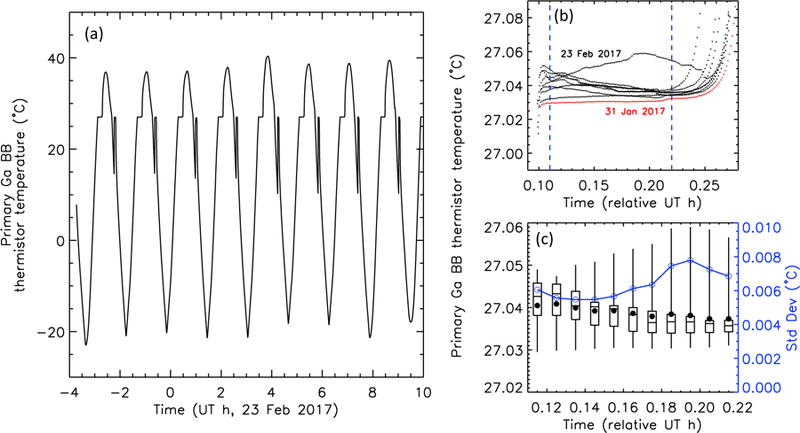
Gallium black body phase transitions (uncontrolled; door open). (**a**) Primary (VACNT) black body–mounted thermistor approximate temperature as a function of time over eight consecutive orbits on 23 February 2017. (**b**) Same eight orbits (black) and a single transition from 31 January 2017 (red), overlaid for comparison. The vertical dashed lines define the phase transition region. (**c**) Statistics for the transitions in (**b**). Box-and-whisker plots indicate the minimum, 25/50/75th percentiles, and maximum values (⦁ is the mean) in each roughly half-minute bin. The blue curve, with axis at right, indicates the corresponding standard deviations.

The RAVAN CubeSat experiences expected temperature oscillations with each orbit. These oscillations can be seen in Figure 9a. This figure shows the temperature as measured by the thermistor bonded to the back of the gallium black body cell on the primary (VACNT) payload door. Please note that the radiometer door is open (normal operations) during this period, and the black body is not being actively controlled—the temperature cycling and phase changes are unforced (also note that the door temperature oscillation is larger than that within the payload body,~20 °C, due to less thermal inertia). The shoulders on the left-hand sides of the oscillations result from the gallium melt and occur over roughly 10 min. The temperature indicated by the thermistor is slightly below the 29.76 °C melting point of gallium. The difference may be explained by a combination of the tolerance of the thermistor absolute temperature accuracy and temperature gradients within the payload door (i.e., the thermistor is not in direct physical contact with the gallium reservoir). The gallium freeze on the righthand side is similar to what was experienced in the laboratory (cf. Figure 7), the temperature falling about 10 °C below the freezing point before briefly spiking back to the freezing point temperature. In any case, we use the gallium melt (not freeze) for the purposes of monitoring radiometer degradation. The repeatability of the gallium melts can be seen in Figure 9b, where the eight orbits shown in Figure 9a are overlaid, along with another melt from several weeks earlier, for comparison. The statistics in Figure 9c show typically consistent behavior, with standard deviations of roughly 0.006°C. One particular orbit in this set deviates from the others at a level of thousandths of a degree. The uncontrolled transitions in Figure 9 are for illustrative purposes, however. Controlled phase transitions are used for calibration, as described next.

**Figure 10:**
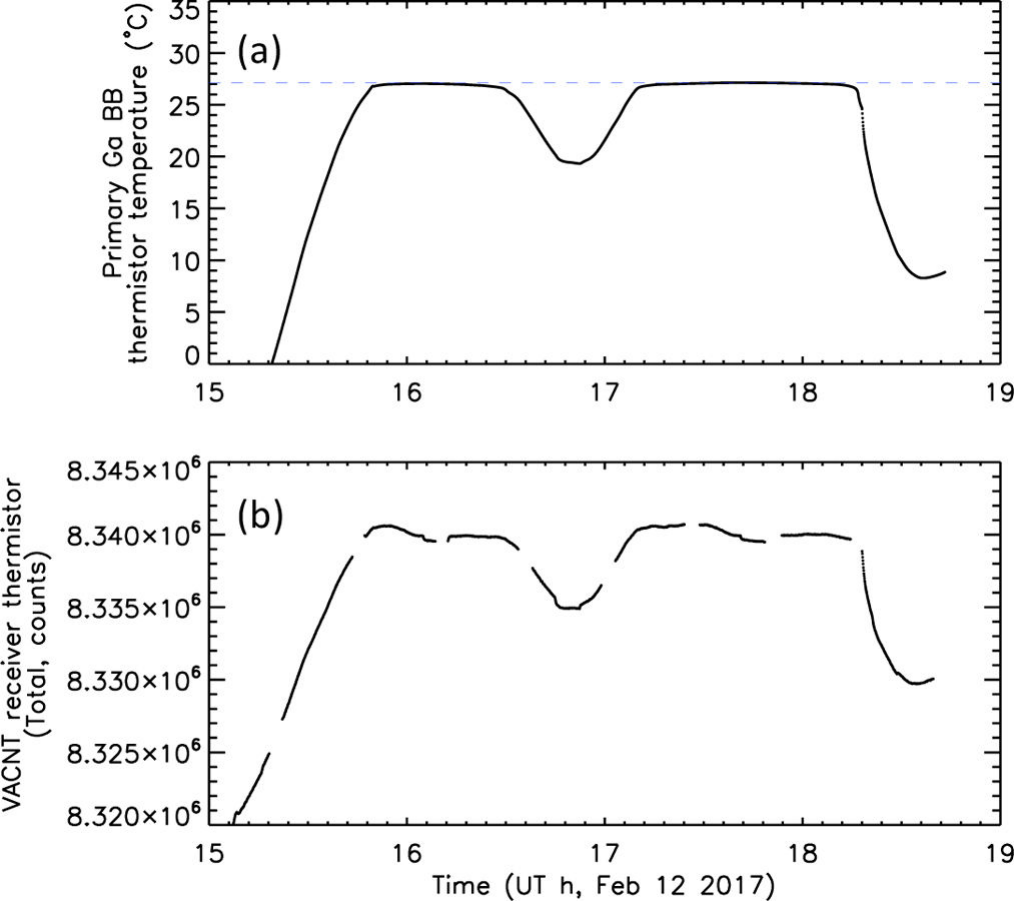
Gallium black body phase transitions (controlled; door closed and black body heater on). (**a**) Primary (VACNT) black body–mounted thermistor approximate temperature as a function of time. The dashed line indicates the temperature measured during the phase transition. (**b**) Corresponding primary (VACNT) radiometer signal. Periods when the calibration heater is on (for internal calibration) have been removed, causing short discontinuities.

To determine the stability of the radiometers using the gallium black bodies, we periodically close the radiometer doors and, using the heaters in the black body assemblies, actively control the gallium melt. In this door-closed configuration, the black body is situated directly above the corresponding Total channel. An example is shown in Figure 10. The heater maintains the cell at the melting point. In this example, the melt is maintained for roughly three hours. The 7 °C cooling in the middle of the period occurs during eclipse, when the spacecraft cools and the black body heater cannot quite generate enough heat to maintain temperature. The radiometer signal is shown in Figure 10b, resembling the temperature profile. The signal level of the stable portions are tracked over time for many such transitions (see Section 3.2.3), and the stable transition is also used as a baseline upon which to perform internal calibrations, where a constant amount of power is input directly into the radiometer absorber heaters, which removes the thermal link, thermistors, and bridge circuit from the calibration.

The black body associated with the primary (VACNT) Total channel failed in March 2017, four months after launch. The thermistor continues to measure temperatures passing through the melting (and freezing) point temperature range, yet no phase transitions are detected. The cause is unknown. The other gallium cell, associated with the secondary (cavity) Total channel, continues to work throughout the entire mission. The two black body cells are in principle identical. Their space environment is somewhat different, however. The primary black body is biased several degrees warmer, and because the primary door is typically open, the primary black body in general experiences wider temperature swings, as it has less benefit from the thermal inertia of the spacecraft.

### 3.2. Radiometers

RAVAN’s four radiometers performed well throughout the mission. They were consistent and stable. In this section, we summarize the key findings and evaluate the strengths and weaknesses of these radiometers—as configured in RAVAN—for making ERB measurements. To do this we examine the radiometer offsets, gains, solar observations, black body measurements, and finally measurements of EOR.

Calibration of the RAVAN radiometers is achieved on orbit with periodic observations of dark space (for offsets) and the Sun (for absolute scale). Table 4 summarizes the symbolic notation used below. The measurement equation for each radiometer relates the receiver thermistor temperature expressed as digital numbers to measured irradiance, *E* (or EOR at spacecraft altitude), which has units of W/m^2^, as follows,

(1) $$E=\left(DN_{\text{rad}}^{⊕}-DN_{\text{offset}}^{⊕}\right)G_{\text{htr}}^{⊕}K_{\text{opt}},$$

where *DN* is the receiver thermistor-bridge analog-to-digital convertor (ADC) counts. The subscripts rad and *offset* correspond to radiometer views of the scene to be measured. Views of the Earth and Sun are denoted *DN*_rad_; views of dark space are denoted *DN*_*offset*_; views of the gallium black bodies are denoted *DN*_BB_. *G*_htr_ is the heater gain—the radiometer response to heating of the receiver. *K*_opt_ is the calibration factor, or optical gain, converting the heater response to absolute optical units. The superscript ⊕ indicates quantities associated with the Earth (nadir) heatsink setpoint.

*K*_opt_ is determined by viewing the Sun,

(2) $$K_{\text{opt}}=\frac{TSI}{\left(DN_{\text{rad}}^{⊙}-DN_{\text{offset}}^{⊙}\right)G_{\text{htr}}^{⊙}},$$

where *TSI* is the total solar irradiance, as measured independently by the SORCE/TIM instrument, and the superscript ⊙ indicates quantities associated with the larger solar heatsink temperature setpoint. During solar views, when *K*_opt_ is measured, it is necessary to increase the heatsink setpoint to maintain control of the heatsinks, which tend to warm up when viewing the Sun. Several different solar setpoints were required throughout the mission. Although empirically determined on orbit with solar views, *K*_opt_ is affected by the aperture area, the absorptivity of the receiver, and the transmission of the sapphire domes (for the SW channels).

Also determined empirically is the heater gain *G*_htr_, which is the measured change in thermistor ADC counts per unit of electrical power applied to the heater during internal calibration. The measurement usually occurs when the radiometer views a stable target such as dark space. The units of *G*_htr_ are completely arbitrary, as the heaters’ purpose is to monitor changes in the radiometer thermal link and temperature readout circuit. What matters is that the applied heat is known in a relative sense. For the measurements presented here, the applied heater power of approximately 1 mW was nearly constant. There are two sets of gains, one for nadir (Earth) viewing and one for solar viewing, $G_{\text{htr}}^{⊕}$ and $G_{\text{htr}}^{⊙}$, respectively, corresponding to the nadir and solar setpoints. Please note that the gain is expected to change significantly with temperature because the sensitivity of the thermistor resistance to temperature is itself a function of temperature. It is assumed in applying Equation (1) that the heatsink temperature is the same during determination of the values G_htr_ and *DN*_offset_ and during radiometric observations (e.g., of the Earth) when *DN*_rad_ is collected. Any change in the heatsink temperature must be associated with an independent determination of *G*_htr_ and *DN*_offset_. By contrast, *K*_opt_ is temperature-independent.

#### 3.2.1. Offsets and Heater Gains

**Figure 11:**
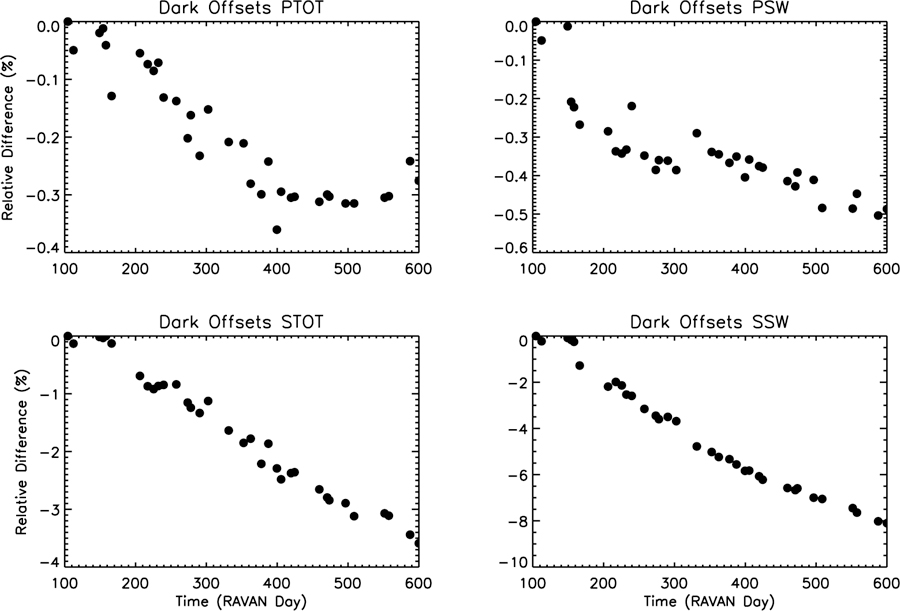
Change in dark space offset measurements over time, normalized to the mean solar response, $\Delta \;DN_{\text{offset}}^{⊕}\times \bar{G_{\text{htr}}^{⊕}}/[\bar{\left(DN_{\text{rad}}^{⊙}-DN_{\text{offset}}^{⊙}\right)}\times \bar{G_{\text{htr}}^{⊙}}]\times \bar{TSI}$, for all four channels as a function of days since RAVAN launch (11 November 2016).

Prerequisites for well-behaved ERB measurements include stable or smoothly varying offsets *DN*_offset_ and heater gains *G*_htr_. Offsets are determined during dark, cold space observations. Results over time for all four radiometers are shown in Figure 11, as defined as follows by

(3) $$\text{offset}\;\text{=}\;DN_{\text{offset}}^{⊕}\times \bar{G_{\text{htr}}^{⊕}}/[\bar{\left(DN_{\text{rad}}^{⊙}-DN_{\text{offset}}^{⊙}\right)}\times \bar{G_{\text{htr}}^{⊙}}]\times \bar{TSI,}$$

which are the offsets over time normalized to the mean solar response. For this figure, mean values of several of the quantities in Equation (3) are used, to isolate offset stability. The long-term behavior is excellent, with the VACNT Total and SW channels changing smoothly by less than 0.4% and 0.5% over the course of the project, respectively. To put these results in a larger context, the RAVAN VACNT Total channel offset drift is comparable to that of the ERBE total channel, while the VACNT SW channel is considerably better than the ERBE SW channel, which drifted approximately 2.3% in two years [24]. The cavity radiometer offsets drift more over time, worse than the RAVAN VACNT and ERBE radiometers, but they do so in a smooth and predictable way.

**Figure 12:**
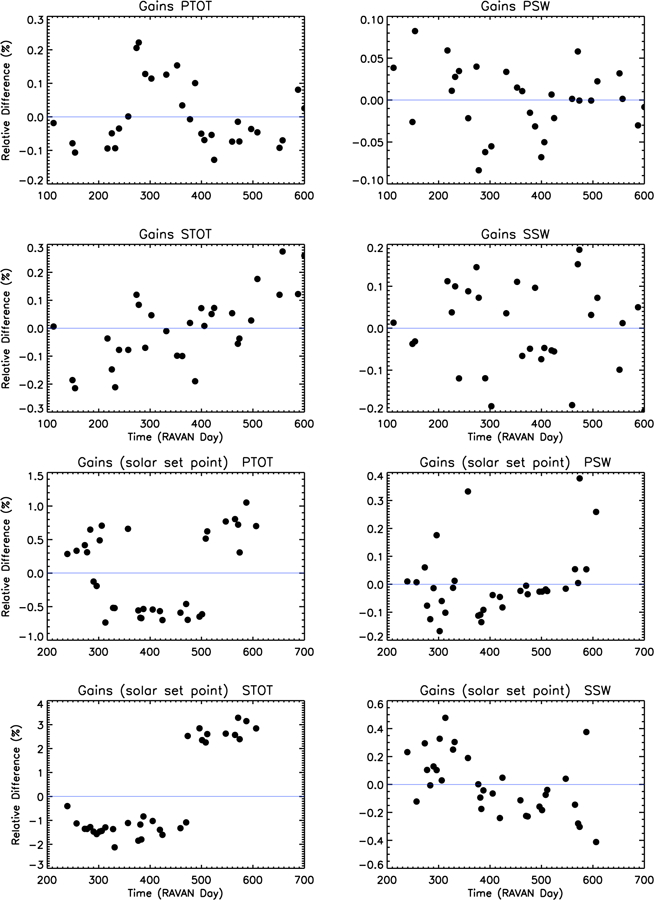
Change in gains at nadir and solar setpoints,$\Delta G_{\text{htr}}^{⊕}$ and $\Delta \;G_{\text{htr}}^{⊙}$ for all four channels. Solar gain data from before RAVAN day 239 have been excluded in these plots, as the heatsink setpoints were still being adjusted and necessarily changing gain values, so the plots show inherent variability at a single setpoint. The exception to this is seen for the STOT solar setpoint gain (at bottom left), where the step discontinuity is due to a change in setpoint value.

The radiometer heater gains are determined as follows. Calibration heaters in the radiometer receivers themselves are used to apply a constant amount of heat (power) directly into the radiometer receivers. The gain relationship is then derived as the radiometer response to this constant input power, which accounts for changes in the thermal link, readout electronics, and thermistor sensitivity. Before the calibration heaters are enabled, the radiometers are placed into a stable state, during either a dark space view (for the solar setpoint gain) or with the payload doors closed (for the nadir setpoint gain, during gallium black body transitions). Gain values are shown in Figure 12. The gains for the most part are stable to within a few tenths of a percent or less. We had to change some of the setpoints at different stages of the mission, to maintain control of the Total channel radiometer heatsinks given the available heater power and spacecraft temperature. This was particularly true at different times of year, as the Earth–Sun distance changed. This caused the occasional discontinuity due to the temperature-dependent sensitivity of the thermistors (e.g., see STOT solar setpoint gains in Figure 12, bottom left).

#### 3.2.2. Solar Measurements

**Figure 13:**
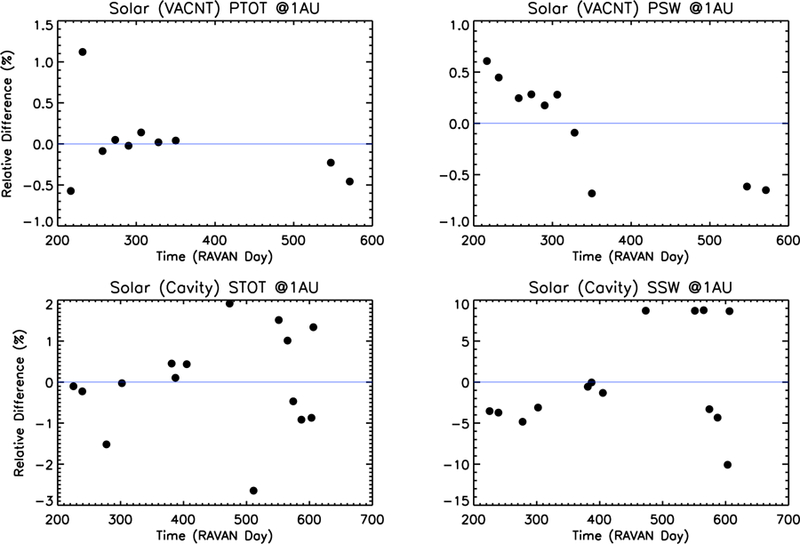
Change in solar measurements, **$\Delta \left(DN_{\text{rad}}^{⊙}-DN_{\text{offset}}^{⊙}\right)\times \;\bar{G_{\text{htr}}^{⊙}}$**, scaled to a mean Earth–Sun distance of 1 AU for all four radiometers as a function of days since RAVAN launch (11 November 2016). The value of $\bar{G_{\text{htr}}^{⊙}}$ is adjusted with time, corresponding to changes in heatsink setpoint. Solar calibrations from before RAVAN day 200 have been excluded in these plots, as early heatsink setpoints were sometimes too low for well-controlled solar calibration.

Solar calibrations are included in our typical calibration sequence. During such, RAVAN stares at the Sun for several minutes, providing enough time for the radiometer readings to stabilize. The solar calibration is another way to assess radiometer long-term stability (in addition to dark space and gallium black body views) as well as a means to set the radiometer absolute scale. Results are shown in Figure 13. The intensity of the solar irradiance reaching the Earth varies annually by roughly 3.5%, owing to the eccentricity of Earth’s orbit about the Sun. The values shown in Figure 13 are scaled to 1 AU, accounting for the Earth–Sun distance. TSI is not actually constant, strictly speaking, yet it varies by less than 0.1% over the course of the 11-year solar cycle, so accounting for this effect is negligible in the plots in Figure 13.

The primary (VACNT) radiometers vary throughout the mission by less than about ±0.5% (excluding one data point). The stability of the secondary (cavity) radiometers during the solar stares is not quite as good but still relatively stable over time.

Despite the good (small and/or smoothly varying) long-term changes in the payload, shorter-term variability may be a challenge for measurements at the level ultimately required for climate accuracy (~0.1%). The short-term variability bears a resemblance to results from the ERBE nonscanner, which was ultimately limited by insufficient thermal knowledge and control [24]. Such knowledge and control are considerably more challenging in a CubeSat, such as RAVAN, that undergoes temperature swings that are approximately an order of magnitude larger than with a traditional large satellite. Nonetheless, there is significant room for improvement for a RAVAN-like instrument (see Section 4).

We conducted a solar calibration maneuver during the solar eclipse of 21 August 2017. The results are shown in Appendix D.

#### 3.2.3. Black Body Measurements

**Figure 14:**
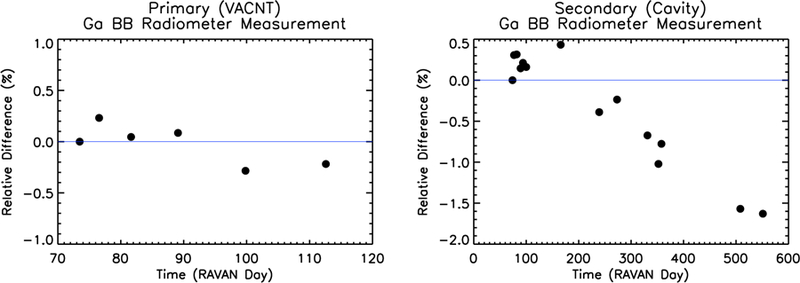
Changes in gallium black body measurements, $\Delta \left(DN_{\text{BB}}^{⊕}-DN_{\text{offset}}^{⊕}\right)\times \;\bar{G_{\text{htr}}^{⊕}}$, for the VACNT and cavity Total channels as a function of days since RAVAN launch (11 November 2016). Please note that the primary black body (left) failed in March 2017.

An assessment of stability may also be found from the gallium black body calibrations, $\left(DN_{\text{BB}}^{⊕}-DN_{\text{offset}}^{⊕}\right)\times \bar{G_{\text{htr}}^{⊗}}$, shown in Figure 14. Based on the presumption that the gallium black body transitions occur at an invariant temperature of the melting point of pure gallium and that the emissivity remains constant, the variability of the infrared measurement during the phase transition is taken to be the variability in the radiometer itself. Over the course of the entire project, the cavity radiometer signal slowly changes by less than 2.5%. The observed change in signal, if from the radiometer, could arise from changes in radiometer offsets associated with closed radiometer doors, which significantly alter the thermal loading on the payload.

#### 3.2.4. Nadir (Earth) Measurements

The ultimate goal of RAVAN-type measurements is the nadir-viewing EOR, *E*, in terms of absolute power units, W/m^2^. Offsets, heater gains, and solar measurements are combined to achieve this, as described above in Equation (1), with the absolute scale provided by the optical gain, *K*_opt_ (see Equation (2)), based on SORCE/TIM.

**Figure 15:**
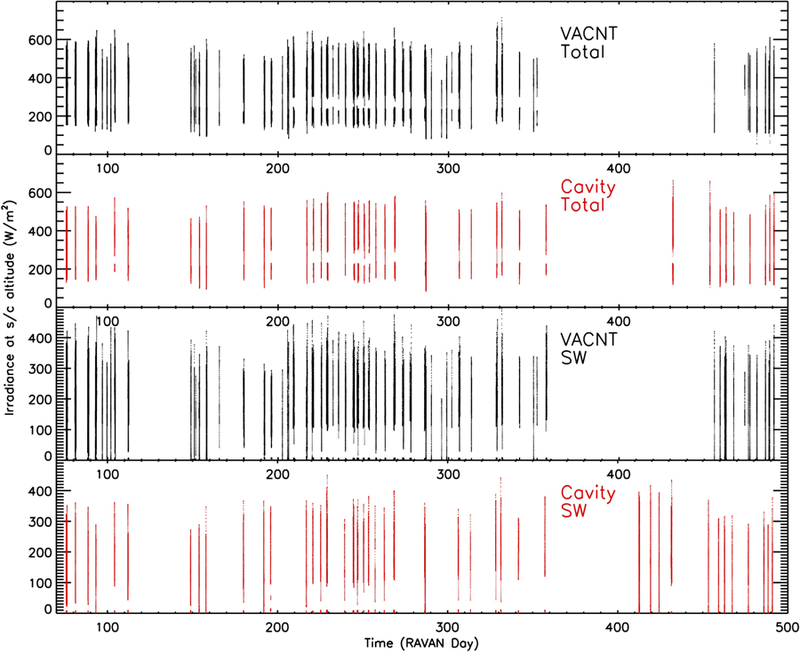
Calibrated Earth outgoing radiation at spacecraft altitude, *E*, from all four radiometers as a function of days since RAVAN launch (11 November 2016). Data gaps are primarily caused by UHF ground interference.

The radiometer doors were opened for the first time on 25 January 2017. Calibrated measurements of EOR at spacecraft altitude are summarized in Figure 15. As designed, RAVAN could collect nadir data continuously, stopping only for calibration sequences (see Table 3), and transmit all the telemetry to the ground. The temporal gaps in the nadir dataset are caused by several technical challenges not related to the payload. The primary factor was UHF ground interference. In practice, we typically collected data 24 h at a time and then transmitted these to the ground over several days. Please note that the ground interference problem could be mitigated in a future mission by using a different radio frequency and/or using multiple ground stations.

An essential part of the evaluation of RAVAN-type data for ERB science is the absolute calibration of the nadir measurements. Our first intention was to compare global monthly mean values of RAVAN EOR with that derived from CERES. However, due to downlink constraints and other limitations mentioned above, the RAVAN dataset is episodic, and simple quantitative monthly mean comparisons are not possible.

**Figure 16:**
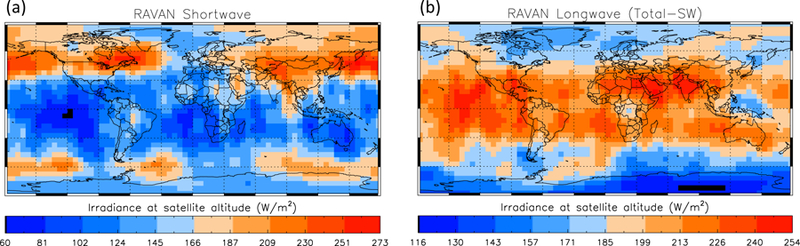
RAVAN Earth outgoing flux measurements (at spacecraft altitude) for the (**a**) SW and (**b**) longwave (mean Total—mean SW) channels, binned into a 5° × 5° latitude–longitude geographic grid for the entire mission, with each WFOV measurement distributed and weighted across the geographic grid.

As a preliminary attempt at CERES comparison—more qualitative in nature—we binned the entire RAVAN nadir irradiance dataset into a 5° × 5° latitude–longitude geographic grid, with each WFOV measurement distributed and weighted across the geographic grid, accounting for RAVAN’s large, 5000-km footprint on the surface. The results are shown in Figure 16. There are two primary caveats associated with this analysis: (1) the RAVAN data are at spacecraft altitude; CERES is referenced to 20 km, and (2) the data are not continuous (see Figure 15), so there is under-sampling and temporal biasing, which could be substantial. However, despite these limitations, there is qualitative agreement between RAVAN and CERES, showing similar latitudinal and even longitudinal patterns (comparison not shown, cf. Dewitte and Clerbaux [7] (Figures 4 and 5)). Several salient features may be noted. High SW reflection and low thermal emission from frontal cloudiness are seen at midlatitudes. There is relatively stronger radiative cooling to the west of the west coasts of South America, southern Africa, Australia, and Mexico, consistent with regions of marine stratocumulus. There is also cooling over the Sahara, Saudi Arabia, and India. For the former two, this is consistent with high surface albedos and clear skies. A more detailed comparison with CERES is beyond the scope of this paper but is the subject of ongoing analysis.

#### 3.2.5. Reconstruction of Spatial Information

Despite being a WFOV nonscanner single-pixel camera, spatial information can be recovered from instruments such as RAVAN. Gristey et al. showed how this can be accomplished with the fitting of high–temporal resolution WFOV data using spherical harmonics [14], following prior work [12,13,34–36]. We have independently developed a similar analysis.

The reconstruction of a global map of TOA flux using RAVAN hinges on the fact that RAVAN’s data is in effect a convolution of TOA flux. RAVAN has an inherent radially symmetric weighting function that determines what proportion of the radiation from any point on Earth reaches the satellite. Regarding both TOA flux and satellite observations as continuous functions of position, RAVAN data are a convolution of the flux function with the weighting function. Recreating the flux from the data is then a matter a deconvolving the data with the weighting function. To take advantage of the mathematical properties of convolutions, we fit RAVAN observations to a sum of spherical harmonic functions and calculate a spherical harmonic decomposition of the weighting function. The final map of TOA flux is then calculated by dividing the spherical harmonic coefficients of RAVAN observations by those of the weighting function and summing the spherical harmonic series with the new coefficients.

The difficulty in this approach is that TOA flux is a function not only of space but of time. Gristey et al. avoided this problem by considering a constellation of 36 RAVAN-like satellites collecting data over a period of an hour and modeling TOA flux as constant over that interval [14]. Our approach instead accounts for the temporal variation in flux by fitting RAVAN observations to a set of products of spherical harmonics with sines and cosines of time. Using a set of basis functions that depends on both space and time allows us to model TOA flux in space and time and to remove the error caused by the static-flux assumption.

**Figure 17:**
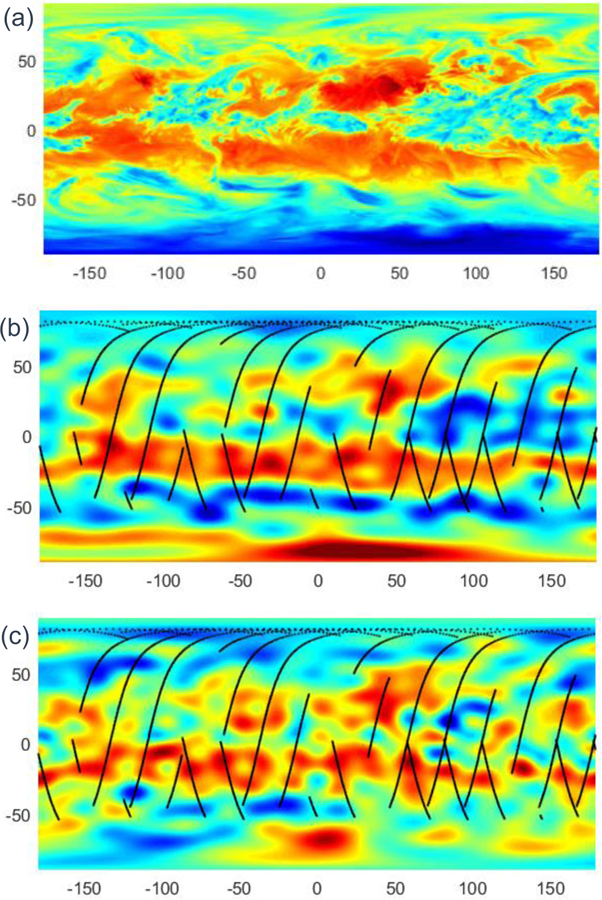
Reconstruction of Earth outgoing longwave irradiance (arbitrary units). (**a**) TOA flux from MERRA-2 reanalysis [37], daily mean for 27 June 2017. (**b**) Spatial recovery for temporally varying MERRA-2 TOA flux with RAVAN sampling for 27 June orbit, shown in black, at spacecraft altitude. (**c**) Spatial recovery of RAVAN longwave (Total–SW) flux data at spacecraft altitude.

An example of our spatial reconstruction of longwave flux for a single day of RAVAN data is shown in Figure 17. We emphasize two points here. First, the basic reconstruction approach in Figure 17b is able to recover synoptic-scale features in the model “truth” in Figure 17a, despite the fact that the radiation field changes throughout the day and with incomplete sampling by RAVAN’s orbit—the model was only sampled where RAVAN data are available, which is not continuous. Second, the flux recovered from the limited RAVAN measurements themselves has features similar to the model and model recovery. This bodes well for the use of simple WFOV radiometers for spatially resolved Earth radiation studies at scales of 100 s of kilometers. This is coarser than that achievable from NFOV radiometers and represents a trade-off depending on science requirements.

Figure 17 shows the LW reconstruction only. Reconstruction of SW flux (not shown) is more challenging. This is because the SW field has greater spatial and temporal variability, and it is distributed less evenly around the globe. For example, sharp edges in albedo (e.g., clouds) are intricate and evolve quickly. The SW field also has an abrupt step function at the day–night terminator. WFOVs straddling the terminator are illuminated only on the day side, potentially creating a “twilight flux” problem for the retrieval of TOA flux from WFOV data [24]. It has been shown, however, that a constellation of (non-Sun-synchronous) satellites can resolve the SW flux field, leading to recovery errors comparable to that in the LW given a sufficient number of satellites [14].

#### 3.2.6. VACNT vs. Cavity Radiometers

**Figure 18:**
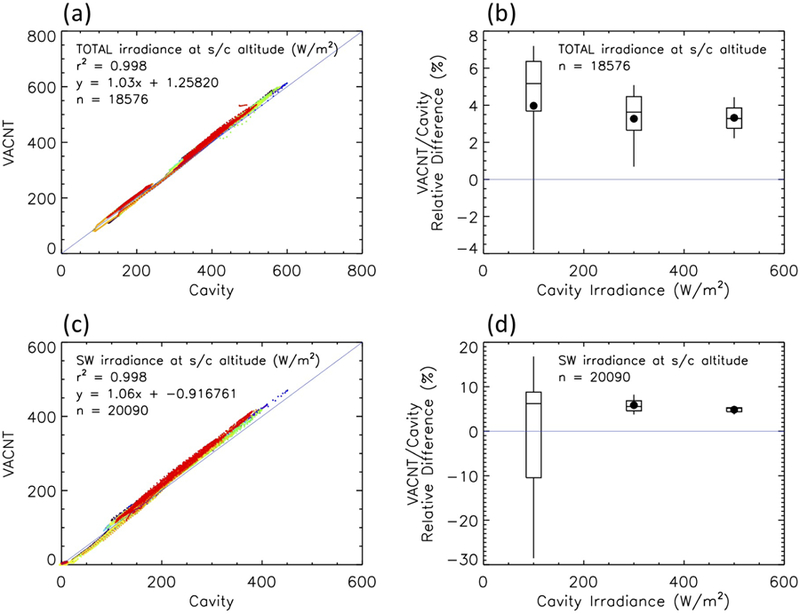
Radiometer intercomparison: VACNT vs. cavity. (**a**,**b**) Total channels. (**c**,**d**) SW channels. Box-and-whisker plots indicate the 5/25/50/75/95th percentiles (⦁ is the mean). Data between RAVAN days 200 and 400 are shown, focusing on the period of greatest calibration stability. The data points are color-coded by time, with earlier times more blue and later times more red.

Intercomparison of the VACNT and cavity radiometers may provide insights into the performance of the VACNTs. Focusing on periods of good data and when all four radiometers were operating, Figure 18 shows the overall excellent correlation found between the new (VACNT) and old (cavity) technologies, with *r*^2^ values of greater than 0.99. Linear fits of the comparisons indicate that on average, the VACNT Total channel is about 3% higher than the cavity Total; the VACNT SW channel is about 6% higher than the cavity SW. Data between RAVAN days 200 and 400 are shown here, focusing on the period of greatest calibration stability. Solar calibrations from earlier in the mission are not as well controlled, and there are fewer in general later in the mission. The VACNT–cavity correlation is similarly good earlier and later, but there are shifts in the relative measurements of TOA flux that change with time (not shown). The proximate cause is the temporal variation of the calibration parameters that go into the absolute nadir calibration (see Equation (1) and Figures 11–13). This will be discussed further in Section 4. Figure 18b,d show the VACNT/cavity relative differences at different levels of TOA flux.

## 4. Discussion

Beyond the primary technology demonstration purpose of the mission, RAVAN represents a benchmark for making Earth radiation budget measurements from a CubeSat. A consideration of the short- and long-term stability of its measurements has important implications for what future improvements would be needed to achieve climate-quality measurements: on the order of tenths of a W/m^2^ (or about 0.1% of the roughly 340 W/m^2^ EOR signal). The offsets of the VACNT radiometer measurements showed good stability, well within the range needed. The gain values were also stable over the course of the mission, with shorter-term variability on the order of 0.1% (slightly larger for the solar setpoint). The radiometer measurements of the gallium black bodies had similar results. Solar observations made with the VACNT Total channel had short-term variations at the 0.1% level; the VACNT SW channel had similar short-term variability but may have shown some degradation over time. The solar measurements with the cavity radiometers varied more over short time periods. The reason for their worse performance than the VACNTs is not readily apparent.

The differences between the VACNT and cavity radiometers during inter-calibrations also set limits on the absolute accuracy of the radiometers (VACNT, cavity, or both). Biases of 3% and 6% for the Total and SW channels, respectively, are much larger than the requirements for climate accuracy. Several differences between the VACNT and cavity radiometers may contribute to the biases, due to: glint from a structure on the payload, offset changes induced by solar heating of the payload (which can, for example, affect the electronics or induce gradients in the heatsink), spectral responsivity (relative spectral differences when comparing absolute solar irradiance and Earth outgoing flux), and degradation. Further analysis and experimentation are needed to understand the large differences. These biases as well as comparisons with CERES are under evaluation.

Although RAVAN has excellent long-term stability, its shorter-term variability may be a challenge for measurements at the level ultimately required for climate accuracy. The short-term variability is not unlike the results from the ERBE nonscanner, which was ultimately limited by insufficient thermal knowledge and control [24]. Such knowledge and control are considerably more challenging in a CubeSat.

There are significant improvements that could be made to future RAVAN-type sensors. Many compromises were made in squeezing four very WFOV radiometers inside such a small, 1U payload volume with limited power resources. One major sacrifice was thermal control of the radiometers and the associated electronics. There was neither room nor power available for the desired level of temperature control of the electronics or the structures around the radiometers. Temperature variation of the RAVAN payload throughout an orbit is approximately 20 °C, or about an order of magnitude larger than with a traditional larger instrument. Such a large temperature swing can induce changes in radiometer offsets arising from the temperature sensitivity of the electronics and from varying temperature gradients within the radiometers. Better active and passive temperature stabilization is possible on a larger bus, and this would have to be addressed when flown on a small spacecraft to achieve the desired level of accuracy.

The radiometer baffles could also be improved. The extremely wide field of view and limited space along the optical axis in RAVAN necessitated shallow and large-diameter baffles with an even larger field of regard. With more space afforded by an even modestly larger small satellite bus and/or fewer radiometers in close proximity, these baffles could be made deeper, which would help to minimize the glint associated with passing out of eclipse. Radiometers built with a narrower field of view, such as CERES and scanner radiometers in general, would allow for smaller baffles and better thermal control of them. Additionally, added space along the optical axis would allow the doors to have a more robust gallium black body: one with a cavity emitter and a longer melting transition.

RAVAN’s short-term variability in offsets, gains, and solar observations (and presumably nadir observations) at a level greater than desirable would be mitigated by more frequent calibration. UHF interference often limited how often we could make observations, but capturing and understanding shorter-term changes would enable us to account for more payload variability.

Finally, an extensive pre-launch calibration, such as described in Section 2, would be a requirement to achieve climate accuracy in a future RAVAN-like science mission.

## 5. Conclusions

The RAVAN project successfully demonstrated the use of vertically aligned carbon nanotubes as broadband radiometer absorbers and gallium phase-change black body cells on a 3U CubeSat. RAVAN was launched 11 November 2016, and its payload operated almost continuously until August 2018, when the technology demonstration ended.

Starting at first light in January 2017, RAVAN made periodic observations of the Earth outgoing irradiance, the Sun, and deep space, with its four radiometers: Total and shortwave channels based on VACNT and cavity absorber technologies. In addition, RAVAN also employed observations of its internal gallium black bodies to monitor long-term radiometer drift. Although RAVAN could make continuous Earth observations as it was designed, technical challenges not associated with the payload, including local UHF ground interference, rendered this impossible. RAVAN observations were episodic, typically with 24 h of measurements followed by several days of telemetry downlink. This did not hinder the primary technology demonstration unduly. Apart from the failure of one of the gallium black bodies after four months, the payload worked well and proved to be radiation-tolerant; the technical difficulties associated with the CubeSat bus could be resolved straightforwardly in a future mission.

Both gallium black bodies underwent phase transitions early in the project. The primary (VACNT-associated) black body failed in March 2017, but the secondary (cavity-associated) black body continued to work as planned. Numerous black body calibrations were performed on orbit, providing an on-orbit calibration reference.

Calibration of the RAVAN radiometers was achieved on orbit with periodic observations of dark space (for offsets) and the Sun (for absolute scale). Prerequisites for well-behaved ERB measurements are stable or smoothly varying offsets and gains. The long-term behavior was excellent, with the VACNT Total and SW channels changing smoothly by less than 0.4% and 0.5% over the course of the project, respectively. The heater gains were stable for both channels at less than a few tenths of a percent. Measurements of the TSI (solar views) were stable and varied by only a few tenths of a percent. A similar assessment of stability can be found from the gallium black body calibrations. Based on the presumption that the gallium black body transitions occur at an invariant temperature of the melting point of pure gallium, the variability of the infrared measurement of the phase transition is taken to be the variability of the radiometer itself. Over the course of the entire project, the cavity radiometer signal has slowly changed by less than 2.5%. Inter-calibration of the VACNT- and cavity-based radiometers viewing the Earth showed the overall good correlation found between the new (VACNT) and old (cavity) technologies, with *r*^2^ values exceeding 0.99 for the Total and SW channels, but with biases of 3% and 6% for the Total and SW channels, respectively, which are much larger than the requirements for climate accuracy (~0.1%). Although RAVAN had excellent long-term stability, its shorter-term variability may also be a challenge for measurements at the level required for climate accuracy. The short-term variability is not unlike the results from the ERBE nonscanner, which was ultimately limited by insufficient thermal knowledge and control [24].

Thermal knowledge and control are considerably more challenging in a small spacecraft. Nevertheless, there is significant room for improvement for a RAVAN-like instrument. Applying greater spacecraft resources to thermal management, more volume for better baffle and black body designs, and extensive pre-launch ground calibration would greatly improve the performance of future RAVAN-type sensors.

An essential part of the evaluation of RAVAN-type data for ERB science is the absolute calibration of the measurements. RAVAN was designed to measure EOR continuously, apart from calibration maneuvers, and our original plan was to compare global monthly mean values of outgoing radiation with that derived from CERES. However, due to downlink constraints and other limitations, the RAVAN dataset is episodic, and simple quantitative monthly mean comparisons are not possible. We made a preliminary qualitative comparison with CERES, and although there are several caveats with this (e.g., sampling bias), the agreement is encouraging.

Continuous measurements of EOR provide an important constraint in climate modeling. Broadband, nonscanner observations from a RAVAN-like platform or its underlying technologies may be a part of the solution to the sustainable ERB challenge. For example, RAVAN technologies enable a constellation mission (either small satellite or hosted payload). If the challenges with this and previous missions [24] can be overcome, the work of Gristey et al. shows how accurate and spatially resolved measurements of ERB can be achieved from a RAVAN-like constellation [14]. The results from RAVAN, while not at the level required for climate measurements, are an important step for the development of science-grade ERB instruments on CubeSat platforms.

## Figures and Tables

**Table 1.** Key RAVAN requirements

Parameter	Requirement
Radiometer absorber	Spectrally flat and stable absorber, reflectivity <0.1%
Sensor head	Response time of 10 s desirable for studying temporal and spatial fluctuations in outgoing radiation; Temperature change of <0.3 mK must be sensed by electronics; Large, 135° FOV to view entire Earth disk, with better than 1° pointing accuracy
Deployable covers	Must clear radiometer 135° FOV; Hundreds of open/close cycles under orbital conditions
Calibration sources (gallium black bodies)	Need repeatability to verify radiometer stability; Temperature repeatability of <50 mK
Electronics and data acquisition	Low-noise resistance bridge to sense changes in radiometer head resistors, short-term stability ~1 ppm; Stable electric heater < 0.1% for radiometer absorber; 24-bit analog-to-digital convertors (ADCs) needed to resolve 0.3 W/m^2^

**Table 2.** RAVAN radiometers.

Symbol	Description	Wavelength Range
PTOT	Primary (VACNT) Total channel	UV–far IR
PSW	Primary (VACNT) SW channel	UV–5.5µm
STOT	Secondary (cavity) Total channel	UV–far IR
SSW	Secondary (cavity) SW channel	UV–5.5µm

**Table 3.** On-orbit calibration modes.

Mode	Configuration	Purpose
Normal	Nadir; VACNT radiometer doors open	Normal Earth data collection
Solar	Point at Sun, doors open	Absolute calibration
Deep space	Point at deep space, doors open	Offset calibration
Ga black body	Doors closed	Calibration stability with gallium black bodies
Internal cal	Doors closed	Gain determination
Inter-calibration	Both doors open	Intercompare VACNT and cavity radiometers

**Table 4.** Calibration nomenclature

Variable	Description
$DN_{\text{rad}}^{⊕}$	Radiometer counts, nadir pointing (nadir setpoint)
$DN_{\text{rad}}^{⊙}$	Radiometer counts, solar stare (solar setpoint)
$DN_{\text{offset}}^{⊕}$	Radiometer counts, dark space (nadir setpoint)
$DN_{\text{offset}}^{⊙}$	Radiometer counts, dark space (solar setpoint)
$DN_{\text{BB}}^{⊕}$	Radiometer counts, black body (nadir setpoint)
$G_{\text{htr}}^{⊕}$	Heater gain (nadir setpoint)
$G_{\text{htr}}^{⊙}$	Heater gain (solar setpoint)
*K*_opt_	Optical gain
*TSI*	Total solar irradiance, measured by SORCE/TIM
*E*	Radiometer measured irradiance (W/m^2^)

**Figure A1:**
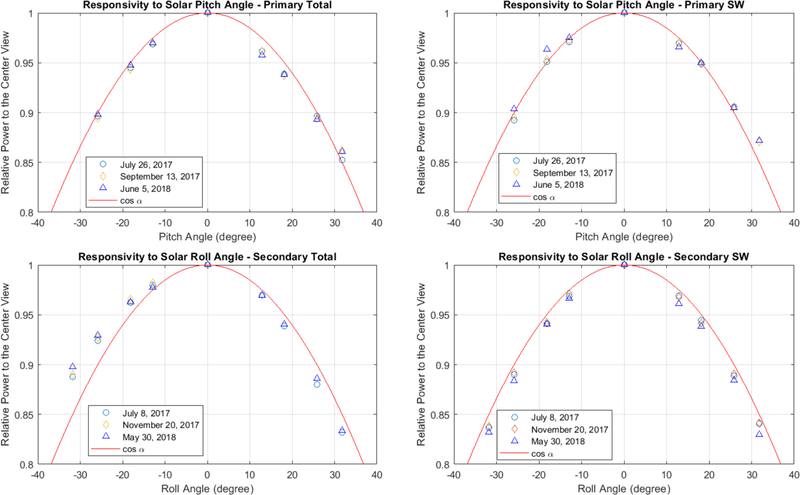
Angular response of the four radiometers as measured on orbit. The relative power was determined by slewing the spacecraft to set angular positions in roll and pitch during solar observations. The ideal cosine response is shown with a red line.

**Figure A2:**
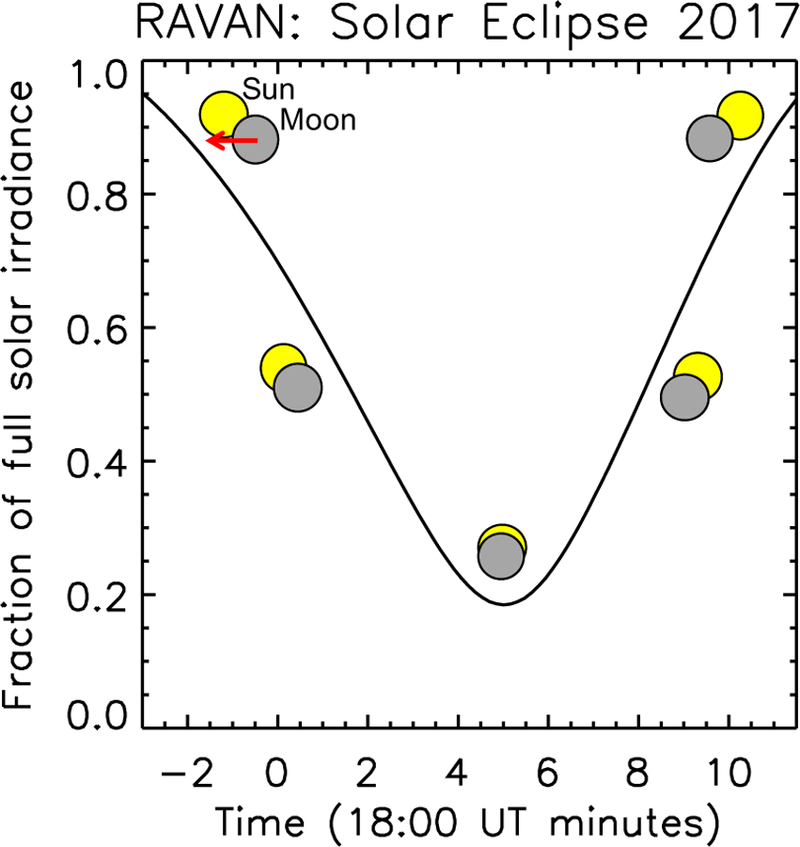
Solar eclipse of 21 August 2017, as viewed by RAVAN’s shortwave VACNT radiometer. The graphics indicate qualitatively the stages of the solar occultation by the Moon during the event. Orbital timing allowed an 80% eclipse at 18:05 UT

## Appendix A. Vertically Aligned Carbon Nanotube Growth Procedure

A silicon substrate is covered by an evaporated barrier layer of 10 nm of Al_2_O_3_ and then 2 nm of iron catalyst. The substrate is then placed in a 2-inch tube furnace that is heated at 750 °C and has an argon/hydrogen flow of 500/250 standard cubic centimeters per minute (sccm). The thermal chemical vapor deposition process is assisted with water vapor by means of a small 10-sccm argon flow through a bubbler, resulting in 100–150 ppm of water vapor in the flow. After a 5-min dwell the samples are exposed to ethylene (the carbon source) at a flow rate of 200 sccm for the prescribed growth time, depending on the height of the CNTs desired. After a set amount of growth time the ethylene flow is stopped, and the sample is left to soak in the furnace for an additional 10 min before being removed. The insertion and removal rates of the sample from the furnace are approximately 1 inch per second. To increase the absorptivity of the VACNTs an oxygen plasma treatment is performed on the sample after it is grown. The 1-mm VACNT forests used in RAVAN took roughly 20 min of exposure, with a total run time of about 45 min. Finally, gold is evaporated onto the back of the silicon substrate to enable soldering in the radiometer assembly.

## Appendix B. Radiometer Temperature Sensing and Electronics

Temperature sensing in RAVAN is performed using thermistors whose resistance is sensed using Wheatstone bridge circuits together with high-resolution (24-bit) sigma–delta ADCs, AD7714. The AD7714 contains a low-noise differential amplifier and multiplexer, which reduces the needed number of amplifiers. Aside from low noise, the main requirement of the temperature sensing electronics is short-term stability, as periodic calibration with the reference heaters removes slow drifts over time. The advantage of a bridge configuration is that it rejects drifts (and noise) in the excitation voltage by directly comparing the thermistors with much more stable reference resistors. Sources of short-term drift arise from the temperature changes of the resistors and ADC during an orbit. To minimize these effects resistors with low temperature coefficients are used, and the AD7714 undergoes frequent internal gain and offset measurements. Radiation testing has demonstrated that the AD7714 continues to operate with minimal changes in performance after exposure to radiation doses of up to 10 krad (Si). However, it is susceptible to destructive latch-up events, so a protection circuit has been implemented. The protection circuit disables the analog power supply regulators when the current draw exceeds approximately 100 mA. The circuit is re-enabled by sending a command to the payload processor. Please note that no latch-up events were ever detected in the RAVAN payload on orbit.

To better maintain long-term stability of the radiometers, there are heaters on each receiver that monitor the radiometer response to absorbed radiation. Use of the heaters accounts for changes in the thermal link between the absorber and heatsink, the temperature dependence of the thermistor resistance, and changes in the thermistor readout electronics. The heaters do not detect changes in the absorber efficiency or transmission of the sapphire domes—those changes are monitored instead by looking at radiometric sources such as the Sun and the black bodies (black bodies for the Total channels). The heaters are driven by 12-bit digital-to-analog converters (DACs), AD7564, used together with an op-amp, OP462. The absorber heaters are resistors that are driven directly with the op-amp output, giving a maximum power per heater of about 4 mW. The voltage across the heaters is sensed using an AD7714 and a precision 2.5-volt reference, AD580, which is connected to one of the AD7714 inputs. Stability of the voltage reference and heater resistors is critical, but their absolute values are not. During radiation testing the AD580 has been observed to degrade by less than 0.05% at 40 krad (Si), which is about 5 times the expected dose per year in RAVAN.

## Appendix C. Radiometer Angular Response

In addition to the solar stare maneuvers used for solar (absolute) calibration, we also slew the spacecraft across the spacecraft–Sun vector to measure the angular response of the radiometers on orbit using the Sun as the source. Examples of the angular response function for each of the four radiometers is shown in Figure A1. The radiometers largely follow an idealized cosine response, which we assumed in our analysis. There are significant deviations from a perfect cosine behavior, most notably in the STOT channel; however, this may not accurately reflect the actual angular response of the radiometers. The solar slew maneuver takes nearly a half hour, and during that time of direct solar exposure it was difficult to maintain good thermal control. In other words, the deviation from the cosine curve may result from a lack of radiometer thermal control, not necessarily optical performance. A detailed laboratory characterization, such as we had to eliminate to meet our accelerated launch schedule (see Section 2), would be needed in a future science mission to measure EOR with the desired accuracy.

## Appendix D. Solar Eclipse Measurements

On 21 August 2017, much of North America experienced a solar eclipse. RAVAN’s orbit fortuitously brought the spacecraft across the U.S. such that it crossed the path of the eclipse at nearly the same time as the Moon’s shadow. By performing an otherwise typical solar calibration during the eclipse, RAVAN’s VACNT radiometers were able to capture the event, as shown for the SW channel in Figure A2. The timing allowed for an 80% total solar eclipse. The significance of the event for RAVAN is that it demonstrates the rapid response of the radiometers to changes—in this case very large changes—in input flux.
